# Exploring the Potential of Non-Coding RNAs as Liquid Biopsy Biomarkers for Lung Cancer Screening: A Literature Review

**DOI:** 10.3390/cancers15194774

**Published:** 2023-09-28

**Authors:** Edoardo Garbo, Benedetta Del Rio, Giorgia Ferrari, Massimiliano Cani, Valerio Maria Napoli, Valentina Bertaglia, Enrica Capelletto, Christian Rolfo, Silvia Novello, Francesco Passiglia

**Affiliations:** 1 Department of Oncology, University of Turin, San Luigi Hospital, 10124 Orbassano, Italy; edoardo.garbo@unito.it (E.G.); benedetta.delrio@unito.it (B.D.R.); giorgia.ferrari@unito.it (G.F.); massimiliano.cani@unito.it (M.C.); valeriomaria.napoli@gmail.com (V.M.N.); valentina.bertaglia@gmail.com (V.B.); enrica.capelletto@gmail.com (E.C.); silvia.novello@unito.it (S.N.); 2 Center for Thoracic Oncology, Tisch Cancer Institute, Mount Sinai Health System, Icahn School of Medicine at Mount Sinai, New York, NY 10029, USA; christian.rolfo@mssm.edu

**Keywords:** lung cancer, screening, liquid biopsy, non-coding RNA

## Abstract

**Simple Summary:**

Low-dose CT scan screening will be widely implemented on a large-scale base, aiming to reduce lung cancer related mortality in the high risk smoking population as already reported in multiple trials, in several countries. Recent evidence has suggested that the identification of liquid biopsy biomarkers may improve its accuracy in lung cancer early detection, reducing the false positive rate as well as overdiagnosis issues and potentially addressing one of the major obstacles in the implementation of Low-dose CT scan alone in this context. RNAs, particularly non-coding RNAs, are for sure the most studied and promising circulating biomarkers in this setting.

**Abstract:**

Lung cancer represent the leading cause of cancer mortality, so several efforts have been focused on the development of a screening program. To address the issue of high overdiagnosis and false positive rates associated to LDCT-based screening, there is a need for new diagnostic biomarkers, with liquid biopsy ncRNAs detection emerging as a promising approach. In this scenario, this work provides an updated summary of the literature evidence about the role of non-coding RNAs in lung cancer screening. A literature search on PubMed was performed including studies which investigated liquid biopsy non-coding RNAs biomarker lung cancer patients and a control cohort. Micro RNAs were the most widely studied biomarkers in this setting but some preliminary evidence was found also for other non-coding RNAs, suggesting that a multi-biomarker based liquid biopsy approach could enhance their efficacy in the screening context. However, further studies are needed in order to optimize detection techniques as well as diagnostic accuracy before introducing novel biomarkers in the early diagnosis setting.

## 1. Introduction

Lung cancer remains nowadays the leading cause of cancer mortality accounting for 12% of overall cancer deaths worldwide. This is certainly linked to the peculiar biological behavior of this disease as well as to a significant diagnostic delay leading to advanced-stage diagnoses in about 50% of cases. For this reason, several efforts over the last years have been focused on the development of effective secondary prevention strategies, with different studies and metanalysis [1] showing that low-dose computed tomography (LDCT) is able to reduce lung cancer-related mortality in high-risk smoking subjects.

In detail, the National Lung Cancer Screening Trial (NLST) and The Dutch-Belgian Randomized Lung Cancer Screening Trial (NELSON) randomized studies demonstrated a significant reduction (around 20%) of lung cancer-related mortality in smoking populations undergoing LDCT as compared to either thorax RX or clinical observation [2,3], leading to the introduction of lung cancer screening in the United States since 2013. Among the different barriers limiting LDCT screening implementation in Europe, the high rate of overdiagnosis and false positive cases represent a relevant unmet need significantly impacting the subject management in real world scenarios. In addition to that, the potential exposure to the imaging radiation and the risk of overtreatment for indolent lung nodules further reduce subjects’ compliance to the LDCT screening. In this context, the integration of tumor biomarkers through liquid biopsy could improve the diagnostic accuracy of LDCT screening in a non-invasive manner aiming to identify the high-risk population requiring further investigation, personalizing screening intervals and likely increasing subjects’ compliance to the screening procedures. Furthermore, the possibility to perform a liquid biopsy in the peripheral hospitals near rural areas could allow to reach a larger smoking population who is usually recalcitrant to the LDCT, thus increasing the access rate to lung cancer screening in a different way and promoting personalized approaches.

A liquid biopsy is able to identify circulating tumor biomarkers that can be considered surrogates of the primary tumor as circulating tumor cells (CTCs), circulating tumor DNA (ctDNA), microRNA (miRNA), and exosomes. Liquid biopsies are already playing an important role in the clinical management of metastatic lung cancer patients through the evaluation of the tumor molecular profiling by ctDNA analysis, while also progressively extending to the early-stage disease in terms of minimal residual disease monitoring as well as cancer interception [4].

The role of CTCs in lung cancer screening has been investigated in several trials since cell dissemination is a relatively early event in tumor progression. These trials showed that CTCs detected in high-risk patients are able to anticipate the diagnosis of lung cancer, even years earlier than CT scans [5]. One of the main problems with using CTCs as a biomarker is represented by their rarity in peripheral blood.

Several studies have investigated the role of CtDNA and cell-free DNA (cfDNA) in screening encountering a fundamental issue: the concentration of cfDNA correlates with the disease burden of the tumor that is very low in early-stage disease, making it difficult to isolate. Despite this limitation, different studies have investigated the role of cfDNA in early diagnosis. To distinguish tumor from non-tumor cfDNA, they have looked at its concentration, genetic changes, or methylation as possible biomarkers [6]. In 2020 a study proposed the use of a cfDNA-based machine-learning method to improve the specificity of LDCT screening with interesting results [7].

Another interesting finding is the development of a blood-based multi-cancer early detection (MCED) test targeting a screening population. The test has been developed for the early detection of more than 50 types of cancer. The MCED test analyzed the methylation patterns of CtDNA and demonstrated high specificity (99.1%) and a positive predicted value of approximately 40% [8]. 

Only a small fraction (approximately 3%) of the genetic transcript is able to encode proteins, while the remaining part is defined as non-coding RNAs (ncRNAs). The definition of “coding” encompasses RNAs that encode proteins from DNA-derived information, such as mRNAs. Noncoding RNAs have a different role since they act as cellular regulators of gene expression at different transcriptional, post-transcriptional, and epigenetic levels. A few exceptions to this definition include some ncRNAs binding ribosomes encoding peptides exerting a modulator function on cellular activities [9]. It has become clear that ncRNAs also play an important role in the communication processes between cancer cells and tumor microenvironment and are crucial for regulating tumor growth [9]. In recent years the knowledge about ncRNAs roles in cancer process has exponentially grown [10], including diagnostic, prognostic, predictive, and therapeutical applications across different cancers and settings [11].

ncRNAs can be classified into two macro-categories: housekeeping ncRNAs and regulatory ncRNAs. Housekeeping ncRNAs regulate basic cellular functions and are ubiquitously expressed. Regulatory ncRNAs play a pivotal role in gene expression regulation and protein translation both at transcriptional and post-transcriptional levels. Increasing evidence points out their role in cancer development, regulation, and growth, making them a precious tool for cancer management at different stages [12,13]. 

Among the housekeeping ncRNAs, we encounter transfer RNA and ribosomal RNA. Regulatory ncRNAs, instead, can be divided into three major groups: circular RNAs (cricRNAs), long noncoding RNAs (>200 nt, lncRNAs), and small noncoding RNAs (<200 nt, sncRNAs) [13]. 

The ncRNAs can be found in different biofluids either as freely or encapsulated in extracellular vesicles [12,14], making them potentially stable biomarkers for clinical use, which have been explored also within LC screening clinical trials [13,15,16].

To date, among the different liquid biopsy biomarkers under clinical investigation in the lung cancer early detection setting, ncRNAs are for sure one of the most promising biomarkers to be implemented in the context of lung cancer screening. For this reason, this review will specifically focus on ncRNA role, describing biological function, available evidence, and clinical trials ongoing in this emerging setting.

## 2. Methods of Literature Search

An extensive literature search was performed on PubMed, using as cut-off date of 16 March 2023. Keywords included: noncoding RNA, lung cancer, screening. A total of 2538 articles were found and screened for eligibility, taking into account three major criteria of inclusion: (1) Single ncRNAs or ncRNA-based genomic signature; (2) biomarker analysis performed on blood or other biofluids; and (3) biomarker involvement in non-small cell lung cancer (NSCLC) screening or early diagnosis.

Both prospective and retrospective studies were considered. Only studies performed on humans were included, but they could include an in vitro/in vivo validation part. Only studies that involved a control group were considered. The control group could also have other pulmonary conditions or pulmonary nodules (PNs) that were retrospectively prospectively identified as benign lesions. Published abstracts without associated full articles were excluded from the analysis. Three independent reviewers collected data from the included articles, and another one subsequently reviewed all of the information.

To draw a clear overview of all clinical trials concerning LC screening that included a liquid biopsy part, we also performed research on clinicaltrials.gov using the following keywords: lung cancer and screening. A total of 601 trials were found and, among them, only those involving LDCT screening and encompassing biofluids collection for biomarkers research were included. 

## 3. Results

### 3.1. Micro RNAs (miRNAs)

MiRNAs are fragments of single-stranded non-coding RNA with a length of approximately 20 ribonucleotides. Since they remain stable in biofluids, unlike other free RNA molecules, they can be detected in both serum and plasma. They regulate gene expression at the post-transcriptional level and are involved in the regulation of cell proliferation and apoptosis. In fact, their targets include oncogenes and tumor suppressor genes and their dysregulation can lead to malignant cell transformation across different tumor types [6,17]. 

miRNAs are one of the pivotal biomarkers explored in phase III trials of lung cancer screening, and our literature search identified studies that used both multi-miRNA signatures (>2 miRNA) and single miRNA approach for early NSCLC detection. In detail both the miR-test, a serum-based 13 miRNA signature, and the micro-RNA signature classifier (MSC), a plasma-based 24 miRNA risk score, showed very promising data for clinical use [18]. 

Overall, 32 studies evaluating the expression of multi-miRNA in early-stage NSCLC patients compared to healthy controls have been identified through our PubMed research. The identified signatures ranged from 2 to 24 miRNAs and were all validated on biological fluids that could be used for liquid biopsies purposes (Table 1). Almost all of the evaluated studies used plasma or serum for miRNA detection and the most used sequencing technique was quantitative real-time PCR (qRT-PCR). Among all of these studies matching our inclusion criteria, only six included a CT scan integrated to a specific miRNA signature for LC diagnosis. 

Sozzi G. et al. [19] conducted a large retrospective analysis in this setting analyzing plasma samples from 939 participants to the Italian randomized MILD LC screening study (69 patients diagnosed with LC and 870 healthy individuals) using a 24-miRNA classifier. They identified a higher sensitivity (87%) and a similar specificity (81%) for LC detection, compared to LDCT alone (79% and 81%, respectively) with a false-positive rate of 3.7% vs. 19.4% with and without MSC integration. 

In another prospective analysis conducted in the BIOMILD study, patients were [16] stratified into four different subgroups based on a miRNA signature classifier (MSC): 2 MSC+ with or without a positive CT scan and 2 MSC− with or without a positive CT scan. Individuals with a positive CT scan and an MSC− had a lower incidence of LC and individuals with both CT and MSC negative had a lower overall LC incidence at four years, interval cancer, stage I, and advanced stages diagnosis, as well as the lowest LC mortality rate at five years as compared to all other subgroups. So, the authors found out that the combined use of LDCT and MSC at baseline was able to predict individual LC incidence and mortality, with a major effect of MSC for LDCT-positive individuals.

Shun J et al. [20] applied a 3 miRNAs (miRs-21, 210, and 486-5p) plasma signature on healthy subjects, patients with benign pulmonary nodules (PNs), and malignant PNs. This approach achieved an area under the curve (AUC) of 0.855 for lung cancer detection in the testing cohort. The panel of the three mi RNA was then validated in an independent cohort of 156 patients who had solitary PNs, and this miRNAs signature produced a 76.32% sensitivity and 85% specificity in differentiating malignant from benign solitary PNs.

The same group [21] screened 10 miRNA differently expressed by LC and healthy smokers sputum and built a logistic regression model on a 2 miRNA combination (miR-31 and miR-210). This model generated an AUC of 0.83 in distinguishing LC patients from healthy smokers, moreover, the combination of CT scans and the 2 miRNA combination achieved an AUC of 0.95. In the validation cohort, the AUC dropped to 0.79, but the combination of miRNA and CT scans improved the specificity and sensitivity compared to CT scan alone.

Another plasma-based approach was conducted by Zheng D. et al. [22], who evaluated circulating small extracellular vesicle (EV) microRNAs in 208 patients with CT-detected PNs. Five miRNAs (let-7b-3p, miR-125b-5p, miR-150-5p, miR-101-3p, and miR-3168), included within the CirsEV-miR model were firstly tested in a small training cohort of 47 patients and then validated in a testing-cohort of 62 patients achieving an AUC for lung cancer detection of 0.920 and 0.760, respectively. This model was then validated in an external cohort of 92 patients (20 patients with benign PNs and 79 with malignant PNs), reaching an AUC of 0.781.

EVs and miRNAs were also tested in this setting by using NGS analysis [23]. They analyzed plasma from patients who had Lung-RADS4 PNs then confirmed as LC, versus over-diagnosed Lung-RADS4 PNs or high-risk Lung-RADS2 screening controls. They identified different expression levels of let-7b-5p, miR-184, and miR-22-3p as biomarkers for potentially discriminating cancer patients from high-risk controls. The multiple logistic regression analyses of the 3 EV miRNAs showed a combined ROC AUC value of 92.4%.

Other pulmonary pathological conditions, such as chronic obstructive pulmonary disease (COPD) and asthma, were included in some of the other studies and represent an interesting approach to eliminate some biases that could be created by smoking-related or pre-existing pulmonary conditions in the implementation of liquid biopsies within lung screening programs. Halvorsen A.R. et al. [24] used serum also from 16 COPD subjects to build their miRNA signature for their prediction model, showing a good performance in discriminating lung cancer from the control groups (AUC 0.89). Yang X et al. [25] used also serum of COPD, in their logistic regression model obtaining not only good performance in discriminating lung cancer patients from controls, but also a higher accuracy for adenocarcinoma (AC) patients rather than squamous cell carcinoma (SCC). Zaporozhchenko I.A. et al. [26] analyzed 179 miRNA in plasma samples obtained from patients with a non-cancerous lung disease (hyper- or metaplastic endobronchitis (EB)) and a cancer-free group of healthy volunteers. They found a 14 miRNA signature discriminating LC group and controls, but interestingly the performance of the model was largely unaffected by the presence of samples from patients with endobronchitis. A similar approach was led by Nadal E. et al. [27] analyzing also serum samples of patients with COPD and identifying a 4 miRNA signature for LC diagnosis, clustering also the discovery set into 2 different groups, characterized by different metastasis-free survival (MFS) and overall survival (OS). Fehlmann T. et al. [15], instead led a large multicenter retrospective cohort study, analyzing 3046 samples of LC patients (including NSCLC and small cell lung cancer, SCLC), and patients with other lung conditions (mostly COPD). A 14-miRNA signature derived from the training set was used to distinguish patients with lung cancer from patients with nontumor lung diseases both in the testing set (accuracy of 92.5%, sensitivity of 96.4%, and specificity of 88.6%) and in the validation set (accuracy of 95.9%, sensitivity of 76.3%, and specificity of 97.5%). 

Some of the studies listed in Table 1, tested another interesting use of LB-based approach in the LC early-diagnosis setting which is LC histological subtype prediction. Lu S. et al. [28] conducted a miRNA analysis on plasma samples of a large cohort of patients (1132 samples, including healthy individuals and patients with NSCLC or SCLC) collected from five medical centers, developing a plasma miRNA panel capable to discriminate LC patients from healthy individuals, and SCLC from NSCLC (AUC 0.878 and 0.869 for training and validation cohort, respectively). Instead, a study by Powrózek T [29], et al., showed that miR-944 had a high diagnostic accuracy for operable squamous cell carcinoma detection (AUC 0.982), whereas miR-3662 for operable adenocarcinoma diagnosis (AUC 0.926). Jiang Y. et al. [30] used an NGS-based approach in analyzing plasma-derived EVs from healthy individuals, patients with early-stage SCLC, and patients with early-stage NSCLC, finding out that miRNA-483-3p derived from plasma EVs could be a potential biomarker for early-stage SCLC diagnosis, while both miRNA-152-3p and miRNA-1277-5p could be used for early-stage NSCLC diagnosis. 

Other efforts of using miRNA-based liquid biopsy for lung cancer early detection were made by using single miRNAs as biomarkers such as miR-17-19 [31], miR-20 [32], miRNA-21 [33,34,35,36,37,38,39,40,41,42], miR-25 [43], miR-29 [44], miR-30 [45], miR-31 [46], miR-125, miR-126 [47,48,49], miR-135 [50], miR-143 [51], miR-145 [32], miR-148/152 family [52], miR-153, miR-155 [36,53,54], miR-182, miR-183 [47], miR-185 [55], miR-184 [56], miR-200 [57], miRNA-210 [47,58], miR-221 [32], miR-223 [32,59], miR-328 [60], miR-339 [61], miR-411 [62], miR-486 [63], miR-499 [64], miR-519 [65], miR-770 [66], mi-R762 [67], microRNA-2355 [68], hsa-miR2116, hsa-miR449c and hsa-miR2117 [69]. These single biomarker-based approaches led to similar results, but the heterogeneity of the study and the lack of a validation cohort make them likely less reliable for clinical implementation in the real-world setting.

**Table 1:** Multiparametric miRNAs studies.

Title	No. of Patients (LC vs. Others)	AUC	CT Combined	External Validation	Biofluid Used	miRNA
Circulating MicroRNAs as Non-Invasive Biomarkers for Early Detection of Non-Small-Cell Lung Cancer [70]	100 vs. 100	0.92 (95% CI: 0.87–0.95)	No	No	Plasma	24 miRNA signature
MicroRNA-based biomarkers for diagnosis of non-small cell lung cancer (NSCLC) [71]	76 vs. 72	0.91 (95% CI: 0.864–0.956)	No	Yes	Plasma and sputum	2 miRNAs (miRs-31-5p and 210-3p) in sputum + 3 miRNAs (miRs-21-5p, 210-3p, and 486-5p)
A unique set of 6 circulating microRNAs for early detection of non-small cell lung cancer [24]	38 vs. 32	0.89	No	Yes	Plasma	6 miRNA signature (miR-429, miR-205, miR-200b, miR-203, miR-125b and miR-34b)
Serum microRNA Signature Is Capable of Early Diagnosis for Non-Small Cell Lung Cancer [25]	63 vs. 15	0.93	No	No	Serum	5 miRNAs (miR-146b, miR-205, miR-29c, miR-30b, and miR-337)
Application of plasma circulating microRNA-448, 506, 4316, and 4478 analysis for non-invasive diagnosis of lung cancer [72]	90 vs. 85	0.896	No	No	Plasma	4 miRNAs (miRNA-448, 506, 4316, and 4478)
Increased micro-RNA 17, 21, and 192 gene expressions improve early diagnosis in non-small cell lung cancer [73]	60 vs. 30	Unk	No	No	Serum	2 miRNA + gene expression (micro-RNA 17, 21, and 192 gene expressions)
Baseline computed tomography screening and blood microRNA predict lung cancer risk and define adequate intervals in the BioMILD trial [16]	2664 vs. 1445	Unk	Yes	Prospective	Serum	13-miRNA serum signature (MSC)
Identifying circulating miRNA biomarkers for early diagnosis and monitoring of lung cancer [74]	48 vs. 984	0.9865	No	No	Serum	5 miRNA (miR-92, miR-140-5p, miR-331-3p, miR-223, miR-374a)
Profiling of 179 miRNA Expression in Blood Plasma of Lung Cancer Patients and Cancer-Free Individuals [26]	50 vs. 50	0.979	No	Yes	Serum and Plasma	14 miRNA
A novel circulating miRNA-based signature for the early diagnosis and prognosis prediction of non–small-cell lung cancer [75]	125 vs. 100	0.882	No	No	Serum	2 miRNA (miR-942 and serum miR-601)
Early Detection of Lung Cancer in Serum by a Panel of MicroRNA Biomarkers [76]	142 vs. 111	0.936	No	Yes	Serum	3 miRNAs (miR-125a-5p, miR-25, and miR-126)
Clinical Utility of a Plasma-Based miRNA Signature Classifier Within Computed Tomography Lung Cancer Screening: A Correlative MILD Trial Study [19]	86 vs. 870	Unk	Yes	Prospective	Serum	24 miRNA (MSC)
Identification of serum miRNAs by nano-quantum dots microarray as diagnostic biomarkers for early detection of non-small cell lung cancer [77]	164 vs. 112	0.93 (95% CI: 0.88, 0.96)	No	Yes	Serum	12 miRNA
External validation of a panel of plasma microRNA biomarkers for lung cancer [78]	471 vs. 489	0.963 (0.862–0.995)	No	Yes	Plasma	4 miRNA (miRs-126-3p, 145, 210-3p and 205-5p)
Two plasma microRNA panels for diagnosis and subtype discrimination of lung cancer [28]	539 vs. 456	0.873	No	Yes	Plasma	6 microRNAs (miR-17, miR-190b, miR-19a, miR-19b, miR-26b, and miR-375)
Circulating microRNA array (miR-182, 200b and 205) for the early diagnosis and poor prognosis predictor of non-small cell lung cancer [79]	50 vs. 30	0.883	No	No	Serum	3 miRNA (miR-182, miR-200b and miR-205)
Potential circulating miRNA signature for early detection of NSCLC [80]	106 vs. 70	0.804	No	Yes	Serum	2 miRNA (miR-21, miR-141)
A Novel Serum 4-microRNA Signature for Lung Cancer Detection [27]	84 vs. 23	0.993	No	Yes	Serum	4 miRNA (miR-141, miR-200b, miR-193b and miR-301)
Sputum microRNA Biomarkers for Identifying Lung Cancer in Indeterminate Solitary Pulmonary Nodules [81]	203 vs. 210	0.92	No	Yes	Sputum	3 miRNA (miR-21, miR-31, miR-210)
Blood-borne miRNA profile-based diagnostic classifier for lung adenocarcinoma [82]	253 vs. 101	0.991	No	Yes	Serum	20 miRNA classifier
Plasma circulating microRNA-944 and microRNA-3662 as potential histologic type-specific early lung cancer biomarkers [29]	90 vs. 85	0.881	No	No	Plasma	2 miRNA (microRNA-944 and microRNA-3662)
Evaluation of circulating small extracellular vesicle-derived miRNAs as diagnostic biomarkers for differentiating between different pathological types of early lung cancer [30]	25 vs. 24	0.791	No	No	Plasma	2 miRNA (miR-152-3p and miR-1277-5p)
Plasma extracellular vesicle microRNA profiling and the identification of a diagnostic signature for stage I lung adenocarcinoma [83]	254 vs. 206	0.917	No	No	Plasma	4 miRNA (hsa-miR-106b-3p, hsa-miR-125a-5p, hsa-miR-3615, and hsa-miR-450b-5p
Evaluating the Use of Circulating MicroRNA Profiles for Lung Cancer Detection in Symptomatic Patients [15]	606 vs. 2440	0.944	No	No	Serum	14-miRNA
Analysis of MicroRNAs in Sputum to Improve Computed Tomography for Lung Cancer Diagnosis [21]	66 vs. 68	0.83	Yes	Yes	Sputum	2 miRNA (miR-31 and miR-210)
Identification and evaluation of circulating small extracellular vesicle microRNAs as diagnostic biomarkers for patients with indeterminate pulmonary nodules [22]	208 (nodules)	0.920	Yes	Yes	Plasma	CirsEV-miR model (let-7b-3p, miR-125b-5p, miR-150-5p, miR-101-3p, and miR-3168)
Diagnosis of lung cancer in individuals with solitary pulmonary nodules by plasma microRNA biomarkers [20]	108 vs. 142	0.855	Yes	Yes	Plasma	3 miRNAs (miRs-21, 210, and 486-5p)
Combining plasma extracellular vesicle Let-7b-5p, miR-184 and circulating miR-22-3p levels for NSCLC diagnosis and drug resistance prediction [23]	40 (nodules)	0.924	Yes	No	Plasma	3 miRNA (miR-184, and miR-22-3p) + EV (let-7b-5p)
Serum miR-1228-3p and miR-181a-5p as Noninvasive Biomarkers for Non-Small Cell Lung Cancer Diagnosis and Prognosis [84]	50 vs. 30	0.711	No	No	Serum	2 miRNA (miR-1228-3p, miR-181a-5p)
A six-microRNA panel in plasma was identified as a potential biomarker for lung adenocarcinoma diagnosis [85]	141 vs. 124	0.84	No	Yes	Serum	6 miRNA (miR-19b-3p, miR-21-5p, miR-221-3p, miR-409-3p, miR-425-5p and miR-584-5p)
Evaluation of Tumor-Derived Exosomal miRNA as Potential Diagnostic Biomarkers for Early-Stage Non–Small Cell Lung Cancer Using Next-Generation Sequencing [86]	46 vs. 42	0.899	No	No	Plasma	8 miRNA (miR-181-5p, miR-30a-3p, miR-30e-3p miR-361-5p, miR-10b-5p, miR-15b-5p, miR-320b)
Identification of a three-miRNA signature as a blood-borne diagnostic marker for early diagnosis of lung adenocarcinoma [87]	238 vs. 257	0.974	No	No	Plasma	3 miRNAs (miR-532, miR-628-3p and miR-425-3p)

### 3.2. Long Non Coding RNAs (lnc-RNAs)

LncRNAs look quite promising since they have been demonstrated to be stable in biofluids [88,89] and to be frequently dysregulated in NSCLC pathogenesis [90]. According to our literature search, a multi-lncRNA approach was conducted across 4 studies. Gupta C et al. [91], analyzed lncRNAs in the sputum of LC patients and cancer-free individuals demonstrating a good ability in discriminating the two groups through a panel containing SNHG1, H19, and HOTAIR (AUC 0.90). The second multi-lncRNA approach was conducted by Yuan S. et al. [92], who collected 528 plasma samples of patients with either LC, other lung conditions, or healthy volunteers. They identified a 4-lncRNA panel (RMRP, NEAT1, TUG1, and MALAT1) with a high diagnostic value for NSCLC (AUC 0.85 for AC and 0.93 for SCC in the expansion cohort). An alternative approach conducted by Li X et al. [93], aimed to search for lncRNAs in tumor-educated platelet (TEP), where a combined use of linc-GTF2H2-1, RP3-466P17.2, and lnc-ST8SIA4-12 achieved an AUC of 0.895. Ultimately in the analysis by Kamel L.M. Et al [94], the combination of GAS5 and SOX2OT showed an AUC of 0.95 for distinguishing LC patients from healthy controls.

Single lncRNA-based studies were conducted for different lncRNAs obtaining lower performances similar to what has been observed with miRNAs until now [95,96,97,98,99,100,101,102,103,104,105,106,107,108], so limiting any clinical implementation in the real word setting.

### 3.3. Circular-RNAs (Circ-RNAs)

Circ-RNAs can be freely detected in biofluids (plasma and saliva) as well as in exosomes [109], and are aberrantly expressed in early-stage lung adenocarcinoma, making them a good biomarker for LC early detection [110]. Even though Yang X. et al. [111] meta-analysis, comparing circRNAs’ expression in tissue and plasma/serum samples, showed that the diagnostic accuracy of tissue was higher (AUC 0.85 vs. 0.79), other evidence points out in the opposite direction. Falin C. et al. [112] validated a combination of circRNAs (hsa_circ_0001492, hsa_circ_0001346, hsa_circ_0000690, and hsa_circ_0001439) that were significantly upregulated in plasma exosomes of AC patients as compared to healthy controls. Hang D. et al. [113] adopted RNA sequencing (RNA-seq) and qRT-PCR approaches to explore cancer-related circRNAs expression, showing that circFARSA was increased in cancerous tissues, and was more abundant in the plasma of LC patients than controls. Other three circRNAs were tested as potential biomarkers for LC early detection with liquid biopsy showing a good diagnostic accuracy: hsa_circ_0023179 [114], hsa_circ_0006423 [115] and circFOXP1 [116].

### 3.4. Other Non-Coding RNAs and Combined Approaches

For what concerns small-nuclear RNAs we found three studies that tested the differences between LC patients and controls. Köhler J. et al. [117], determined RNU2-1f in the serum of patients with LC, chronic lung disease, and healthy controls, showing the ability to discriminate the LC group from others (AUC of 0.91). Moreover, the two isoforms of RNU2 (RNU2-1 and RNU2-2) were also tested in another study by Mazières J et al. [118], who demonstrated that miR-U2-1 was able to discriminate between patients with COPD and patients with COPD and lung cancer (AUC of 0.866). Dong et al. [119] used a tumor-platelet educated approach, finding out that TEP U1, U2, U5 were decreased in early-stage lung cancer patients compared with those in healthy subjects.

For what concerns piwiRNAs we found a study by Li J. et al. [120] demonstrating that piR-hsa-26925 and piR-hsa-5444 had a significantly higher level in serum exosome samples of AC patients than healthy controls.

No studies matching our inclusion criteria were found about ribosomal RNA (rRNA), transfer RNA (tRNA), and small nucleolar- RNAs (sno-RNAs) in the context of lung cancer screening.

Few studies were conducted using a combined ncRNAs approach, according to our inclusion criteria. In detail Peng H et al. [121] constructed a miRNA and MALAT1 non-coding RNA panel showing a good performance also in detecting stages I/II/III NSCLC. A panel of seven small ncRNA pair ratios was tested by Dou Y. et al. [122] and could differentiate AC patients from other lung diseases of high-risk controls.

### 3.5. Ongoing Clinical Trials on Liquid Biopsy in Lung Cancer Screening

We also performed a study of ongoing clinical trials on clinicaltrials.gov using the keywords “lung cancer” and “screening”. The data collection was completed on 16 March 2023 and identified a total of 601 ongoing trials related to lung cancer screening. Out of the 601 clinical trials identified, we selected 55 trials incorporating liquid biopsy and the analysis of biological samples for the detection of predictive biomarkers in the setting of LC screening (Table 2). The selected trials did not exclusively include healthy individuals at high risk of developing lung cancer, but also those with lung nodules, CT suspicion or pathologically confirmed lung cancer, as well as other benign lung diseases. Furthermore, a particularly noteworthy study included only never-smokers (defined as individuals with a lifetime exposure of less than 100 cigarettes) and Asian women (NCT05164757).

Among the 55 clinical trials shortlisted based on our inclusion criteria, 25 of them involved the use of chest CT or LDCT scans as a diagnostic tool for lung cancer screening. The HANSE trial (NCT04913155) also investigated other indicators such as coronary calcium score and emphysema score. One of the selected studies involved the use of chest MRI to assess the concordance of imaging features of nodules between LDCT and MRI in the study population (NCT05699213). Concluding, a small portion of these studies incorporates pulmonary function testing within their research protocols.

These selected trials also involved the collection and analysis of various biological samples to identify possible biomarkers for the early detection of lung cancer. Specifically, they included blood samples, different airways samples (bronchoalveolar lavage, BAL, bronchial biopsy and brushing samples, nasal swab, and brush samples), sputum samples, buccal swab samples, urine samples, and feces samples. 

Among the 55 clinical trials that met our inclusion criteria, blood samples were collected in 52 trials, but only 33 of these explicitly state the specific biomarkers that were intended to be analyzed, including miRNA, epigenetic biomarkers, circulating free DNA (cfDNA), circulating tumor DNA (ctDNA), circulating tumor cells (CTC), Associated Macrophage-Like cells (CAMLs), exosome antigens, methylation changes in peripheral blood mononuclear cells (PBMC) and circulating tumor DNA, RNA integrity number (RIN), protein signatures, DNA methylation, whole-genome methylation, tumor antibodies, circulating nucleic acids, proteins, and genetic variation single nucleotide polymorphisms (SNPs), as well as DNA and RNA for germline analysis and whole-exome sequencing (WES). Specifically, only eight trials have a clear focus on the identification and analysis of miRNA. Moreover, 3 out of these 52 clinical trials involve the storage of blood samples in biobanks for potential future studies.

A small part of the clinical trials that met our inclusion criteria have already published results. We have already discussed the results of The Multicentric Italian Lung Detection (MILD) study, a prospective randomized controlled screening trial that compared the diagnostic performance of two different LDCT screening intervals in high-risk smoking populations. After a median active screening period of 6.2 years, the MILD trial concluded that biennial LDCT screening for lung cancer in individuals with a negative baseline LDCT can achieve a comparable clinical outcome to annual LDCT screening. The study, as already said, highlights the potential of circulating miRNAs as biomarkers for cancer detection and prognosis [19]. 

In this scenario, we previously illustrated also the results of the BioMILD trial [16] which is a large prospective study that aims to optimize the screening intensity for lung cancer through a combination of LDCT and a blood-based microRNA assay (MSC). The participants underwent baseline LDCT examination, spirometry, and miRNA profiling, and were followed for a median duration of 5.3 years. The study discovered that participants who were double-negative for LDCT and MSC had very low rates of lung cancer incidence and mortality. As a result, they were recommended to undergo LDCT screening once every three years. The results of the study confirmed that the combined use of LDCT and blood miRNAs at baseline can predict individual lung cancer incidence and mortality.

The New York University Lung Cancer Biomarker Center (NYULCBC) enrolled high-risk smokers and lung cancer patients into a screening cohort and a “rule-out lung cancer” cohort with the aim of identifying and validating biomarkers for the early detection of lung cancer. The participants completed a medical and respiratory symptom questionnaire, underwent pulmonary function testing, blood sampling, chest CT, and were followed up for nodule stability. Greenberg et al. [123] conducted a study to evaluate the levels of serum S-Adenosylmethionine (AdoMet) in participants enrolled in the NYULCBC trial from February to August 2004. The study found that patients with lung cancer had higher levels of serum AdoMet compared to healthy non-smokers and high-risk smokers with small noncalcified nodules. AdoMet level alone was able to differentiate patients with lung cancer from smokers with benign nodules with high sensitivity and specificity. When combined with nodule size, AdoMet level showed a sensitivity and specificity of 100% and 94%, respectively. The elevated AdoMet level in lung cancer patients may relate to the role of AdoMet in DNA methylation, as hypermethylation of the promoter regions of tumor suppressor genes in lung cancer and other malignancies has been reported. AdoMet could be a promising marker for early-stage lung cancer detection, but further studies are needed to confirm its efficacy in larger populations and its clinical utility for recurrence diagnosis.

Several clinical trials are investigating the effectiveness of incorporating new blood tests along with LDCT for lung cancer screening. The NCT01925625 trial tested whether using the EarlyCDT-Lung test and subsequent CT scanning to identify individuals at high risk of lung cancer could reduce the incidence of patients with advanced-stage lung cancer at diagnosis compared to standard clinical practice. The EarlyCDT-Lung test used an enzyme-linked immunosorbent assay (ELISA) to measure seven distinct autoantibodies, each having specificity for different tumor-associated antigens, including p53, NY-ESO-1, CAGE, GBU4-5, HuD, MAGE A4 and SOX2. At 2 years, the test showed high specificity (90.4%) and moderate sensitivity (32.1%) with a higher number of early-stage lung cancers detected in the intervention arm. However, no significant differences were observed in lung cancer and all-cause mortality between the intervention and control groups [124]. The study suggested that blood-based biomarkers followed by LDCT can detect early-stage lung cancer, but more research is required to determine the long-term impact and increase engagement. 

Another test is Lung EpiCheck (Nucleix, Modi’in, Israel), which has been designed to detect hypermethylation status across six markers that are associated with lung cancer, by using cfDNA analysis. Recently, this test has been validated in European and Chinese patients samples and has demonstrated high accuracy rates, as well as an independent predictive capability for lung cancer detection, suggesting potential utility for improving screening access and compliance among high-risk populations [125]. In this scenario, the NCT04968548 trial is an observational study aimed at collecting blood samples and clinical data from individuals undergoing LDCT for lung cancer screening and those with confirmed lung cancer to determine and validate the Lung EpiCheck.

Furthermore, the NCT03452514 trial aims to validate the HMBDx microRNA Test by collecting blood samples from 400 individuals who are undergoing LDCT screening. The study plans to analyze microRNA signatures using a novel lung cancer test, compare the results with those obtained through CT scan findings and follow-up tests, and maintain a minimum follow-up period of 12 months post-enrollment. 

Lastly, the primary objective of the NCT05306288 clinical trial is to validate the DELFI-based test for detecting lung cancer among individuals eligible for routine screening, using a genome-wide analysis technique called “DNA evaluation of fragments for early interception” (DELFI) to detect abnormalities in cfDNA [126]. Participants have blood collected and undergo medical record review at baseline and two additional time points. Presently, no conclusions are available as these last two clinical trials are still ongoing.

## 4. Discussion

Given the elevated incidence of overdiagnosis and false positive cases associated with LDCT screening, the identification of reliable biomarkers capable of improving the diagnostic accuracy, represents an unmet need. In this scenario, ncRNAs might be a potential reliable tool to stratify populations into precise categories of lung cancer risk. To date, microRNAs are those most investigated in large prospective trials for lung cancer screening purposes. As reported in the bioMILD trial, the implementation of miRNAs in NSCLC screening can reduce false positive rates and improve diagnostic accuracy of LDCT, thus opening the way for personalized screening approaches. 

Furthermore, what emerged from our literature research is an extreme heterogeneity of the conducted studies using different methodologies of analyses and selecting various risk populations. This inevitably can be seen as a positive aspect, as in most of the studies presented, the results were consistent with the ability of ncRNAs to distinguish populations with lung cancer from those that were negative or might face overdiagnosis if subjected to LDCT. However, from a methodological point of view, it clearly constitutes a major issue to be addressed with further research in order to standardize a potential application of ncRNAs liquid biopsy in a real-world setting and safely implement them into our clinical practice.

Methodological limitations of analysis also emerged from this wide literature search, including the heterogeneity of ncRNA detection methods used across the different studies, mostly based on q-RT-PCR, but also on NGS limited panels, digital-droplet PCR, and RNA-seq, pointed out the issue of standardization methods to make ncRNAs part of clinical practice. In fact, from a practical point of view, detecting and sequencing this genetic material might be challenging for different reasons. Next-generation sequencing (NGS) is one of the high-throughput screening methods that can be implemented more efficiently into clinical research to validate panels of ncRNAs that can be used for LC screening research programs [127]. 

Some limits need also to be considered once we propose ncRNAs as a biofluid-based biomarker for LC screening but also in general for other purposes. First of all, the overall quantity of ncRNAs is generally lower in the intracellular, extracellular ambient, as well as in plasma or serum as compared to other genetic material, so it might be a challenge to detect them in patient-derived blood samples [128]. Another potential issue is related to the post-transcriptional modifications of ncRNA sequence making them similar to other ncRNAs of the same family (such as for micro RNAs, miRNAs), as well as to mRNAs sequence, making it difficult to distinguish each other. Another issue related to the use of ncRNAs liquid biopsy in LC screening context is the cost-effectiveness benefit that the real-world application of these techniques could imply. For now, large studies demonstrated a clear clinical benefit of LDCT-based screening programs [1], but it is not clear if the implementation of LB in this setting will be feasible from this point of view. In addition to that, further clinical trials testing the role of LB ncRNAs detection in non and light-smokers should be conducted.

Moreover, lung cancer heterogeneity is well-known and established across the board [129,130,131], limiting the use of single-biomarker based approaches. Conversely, the use of multiple biomarkers of the same class or multiple ncRNA class panels could improve diagnostic accuracy within screening programs, since the genetic variability among different tumors and individuals could be covered by different biomarkers working together at the same time.

## 5. Conclusions

In conclusion, the implementation of ncRNAs for LC screening purposes is one of the most promising biomarkers to integrate LB in the prevention setting. For this reason, standardization of protocols for LB ncRNAs detection and further prospective clinical trials with larger cohorts are needed to validate and introduce these novel biomarkers in the clinical arena. In addition, we believe that the use of ncRNAs belonging to multiple subcategories can further improve the ability to discriminate between negative and positive subjects, and therefore using expanded ncRNA panels for LC early detection should be one of the next implementations for LB studies in this research context. To date, clinicians should carefully interpret LB results coming from the early diagnosis studies and policy-makers should push research to focus also on the implementation of liquid biopsy in the real-world setting.

## Figures and Tables

**Table 2:** Ongoing clinical trials investigating liquid biopsy non-coding RNA for lung cancer screening.

Trial (ClinicalTrials.gov Identifier/ Name of Study)	Intervention	Description	Posted Results
NCT02247453 (BIOMILD)	Spirometry, blood samples for miRNA profiling	Plasma microRNA Profiling as First Line Screening Test for Lung Cancer Detection: a Prospective Study	Yes
NCT04913155 (HANSE)	Blood samples	HANSE-Holistic Implementation Study Assessing a Northern German Interdisciplinary Lung Cancer Screening Effort, Population-based Screening Study -Prospective, Randomized Comparator Controlled	No
NCT05452200 (ILYAD)	Spirometry, blood and breath samples	Pilot Study on Lung Cancer Screening Implementation Among Employees at Lyon Hospital	No
NCT02777996 (ITALUNG)	Blood and sputum samples	An Italian Randomized Trial for the Evaluation of the Efficacy of Lung Cancer Screening with Low Dose Computed Tomography	Yes
NCT05494021 (CLUS 3.0)	Blood samples	Lung Cancer Screening with Low-dose CT in China (CLUS Study) Version 3.0	No
NCT00103363	Sputum sample for cytology, spirometry, blood samples	Sputum Cytology in Screening Heavy Smokers for Lung Cancer	No
NCT03975504 (CLUS 2.0)	Blood samples	Community-based Lung Cancer Screening with Low-dose CT in China (CLUS Study) Version 2.0	No
NCT05384769	Liquid biopsy	Feasibility Study of Lung Cancer Screening Using Cell-Free DNA Liquid Biopsy at Home in High-Risk Current and Former Smokers	No
NCT00625690	Blood samples	Development of a Lung Cancer-Screening Program at the University of Nebraska Medical Center: A Feasibility Study	No
NCT04968548	Blood sample tested with Lung EpiCheck (Nucleix)	Determination and Validation of Lung EpiCheck^®^: A Multianalyte Assay for Lung Cancer Prediction. A Case-Control Study	No
NCT01687647 (AMORCE-CBP)	Blood and sputum samples	Interest of Morphometric Analysis of Sputum Cytology for Lung Cancer Screening in Workers Highly Exposed to Asbestos-Exploratory Analysis of Biomarkers Predictive for Lung Cancer	No
NCT03452514	Blood samples for miRNA profiling	Prospective Longitudinal Blinded Observational Diagnostic Study-Addition of microRNA Blood Test to Lung Cancer Screening Low Dose CT	No
NCT05174468	Breath samples	Analysis of Volatile Chemicals in Lung Cancer Screen-Eligible Subjects Using Infrared Spectroscopy	No
NCT00301119 (NYULCBC)	Blood and sputum samples, urine, BAL, lung tissue, buccal swab	Lung Cancer Biomarkers and Screening	No
NCT05306288 (DELFI-L201)	Blood samples for ctDNA detection using a DELFI-based test	Cancer Screening Assay Using DELFI; A Clinical Validation Study in Lung	No
NCT04204499 (ASCENT)	Blood samples for DNA/RNA germline analysis, tumor surplus collected for WES and RNA profiling	Analysis of Screen-detected Lung Cancers’ Genomic Traits	No
NCT02500693 (AIR)	Blood samples for CTC analysis	Circulating Tumor Cells and Early Diagnosis of Lung Cancer in Patients with Chronic Obstructive Pulmonary Disease	No
NCT02611570	Blood samples, urine	Low Dose Computed Tomography Screening Study in Non-smokers with Risk Factors for Lung Cancer in Taiwan	No
NCT04315753	Blood samples for CTC analysis, exosome antigens and cfDNA mutational analysis	Circulating and Imaging Biomarkers to Improve Lung Cancer Management and Early Detection	No
NCT04409444 (qUEST)	Blood sample for analysis of CTC, circulating nucleic acid, proteins and genetic variations (SNPs); nasal swab and brush samples for the identification of inflammatory markers	An Observational Cohort Study Investigating the Impact of Community-based Lung Cancer Screening Across a Deprived Geographical Area and the Role of Biomarkers for the Early Detection of Lung Cancer	No
NCT05432128	Molecular typing early lung cancer, blood samples for peripheral blood ctDNA methylation testing	Molecular Typing System for Early Screening and Diagnosis of Lung Cancer Combined with Liquid Biopsy Technology	No
NCT00512746	Blood samples and sputum samples for cytology and cytometry	A Randomised Controlled Trial of Surveillance for the Early Detection of Lung Cancer in an at Risk Group	No
NCT01475500	Blood and sputum samples, urine, nasal washings, buccal epithelium, endobronchial tissue, and BAL	Nashville Early Diagnosis Lung Cancer Project	No
NCT00420862 (DANTE Trial)	Blood and sputum samples	A Randomized Study on Lung Cancer Screening with Low-Dose Spiral Computed Tomography	Yes
NCT02504697 (DECAMP-2)	Blood, sputum and airway samples, urine	Detection of Early Lung Cancer Among Military Personnel Study 2 (DECAMP-2): Screening of Patients with Early Stage Lung Cancer or at High Risk for Developing Lung Cancer	No
NCT00899262 (MEDLUNG)	Sputum samples and endobronchial biopsy tissue specimens	Biomarkers for Early Detection of Lung Cancer in Patients with Lung Cancer, Participants at High-Risk for Developing Lung Cancer, or Healthy Volunteers	No
NCT05164757	Blood samples	New York Female Asian Nonsmoker Screening Study	No
NCT02837809 (MILD)	Blood samples for ctDNA detection and miRNA profiling	Early Lung Cancer Detection with Spiral Computed Tomography (CT), Positron Emission Tomography (PET) and Biomarkers: Randomized Trial in High Risk Individuals	Yes
NCT03628638	Blood samples for protein and nucleic acids analysis	Blood Sample Collection in Subjects Participating in a Lung Cancer Screening Program	No
NCT03934866 (SUMMIT)	Blood samples for cfNA analysis	The SUMMIT Study: Cancer Screening Study with or Without Low Dose Lung CT to Validate a Multi-cancer Early Detection Test	No
NCT03499678	Blood samples for the analysis of methylation changes in PBMC and ctDNA	Clinical Trials on Detection of Lung Cancer with Non-invasive Method Based on DNA Methylation of Circulated Tumor DNA, PBMC and T Cells	No
NCT01982149	Blood samples, urine, and airway epithelium	Incorporation of Genetic Expression of Airway Epithelium with CT Screening for Lung Cancer	No
NCT03181256	Blood samples, sputum, urine, nasal and buccal epithelium	Early Detection of Lung Cancer in the Medically Underserved Population	No
NCT04165564 (DECAMP 1 PLUS)	Blood and airway samples	DECAMP 1 PLUS: Prediction of Lung Cancer Using Noninvasive Biomarkers	No
NCT04957433	Blood and sputum samples	Lung Health Check Biomarker Study	No
NCT04558255	Blood samples	Plasma Biomarkers as a Non-invasive Approach for Early Diagnosis of Lung Cancer	No
NCT04323579 (CLEARLY)	Blood samples for the analysis of CTs, miRNA signatures, exosome antigens and protein signatures	Validation of Multiparametric Models and Circulating and Imaging Biomarkers to Improve Lung Cancer EARLY Detection	No
NCT05462795	Blood samples for DNA methylation analysis	Liquid Biopsy to Distinguish Malignant from Benign Pulmonary Nodules and to Monitor Response to Therapy	No
NCT03791034	Blood samples for cfDNA analysis	Prospective Feasibility Study of Cell Free Circulating Tumor DNA for the Diagnosis and Treatment Monitoring in Early-stage Non-small Cell Lung Cancer	No
NCT04216511 (ECLC)	Blood samples for the detection of tumor autoantibody	Clinic Validation of Autoantibody Panel for Lung Cancer Diagnosis in Chinese Population	No
NCT03633006	Blood samples	Blood Sample Collection in Subjects with Pulmonary Nodules or CT Suspicion of Lung Cancer or Pathologically Diagnosed Lung Cancer	No
NCT01925625 (ECLS)	Blood samples for autoantibodies detection using the EarlyCDT-Lung Test	Detection in Blood of Autoantibodies to Tumour Antigens as a Case-finding Method in Lung Cancer Using the EarlyCDT-Lung Test	No
NCT04825834 (DELFI-L101)	Blood samples for DNA based biomarkers analysis using the DELFI assay	DNA Evaluation of Fragments for Early Interception-Lung Cancer Training Study	No
NCT04156360	Blood samples for the detection of CTCs and CAMLs	Construction and Evaluation of the Liquid Biopsy-based Early Diagnostic Model for Lung Cancer	No
NCT04462185	Blood samples and nasal epithelium	A Prospective Cohort Study of Chinese Patients with Pulmonary Nodules: Prediction of Lung Cancer Using Noninvasive Biomarkers	No
NCT03293433	Blood samples for miRNA analysis	Quantification of microRNAs in Diagnosis of Pulmonary Nodules: Reproducibility Analysis of Intra- and Inter-observer and Inter-laboratory: Project miR-Nod	No
NCT03685669	Blood samples for ctDNA methylation analysis	Detection of Early-stage Lung Cancer by Using Methylation Signatures in Circulating Tumor DNA	No
NCT05227261 (K-DETEK)	Blood samples for ctDNA analysis	Assessment of a Novel Blood Test in Early Detection of the Five Common Cancers Based on the Investigation of the Circulating Tumour DNA	No
NCT03181490	Blood samples for ctDNA methylation profiles analysis	Multi-centers Validation of a Circulating Tumor DNA Assay to Differentiate Benign and Malignant Pulmonary Nodules Via Targeted High-throughput DNA Methylation Sequencing	No
NCT05415670	Blood sample for whole-genome methylation sequencing	Development a Pulmonary Nodules Diagnosis Classification Model for Benign/Malignant of Bronchoscopic Biopsy Specimens Based on High-throughput Whole-genome Methylation Sequencing (GM-seq)	No
NCT03989219	Blood samples for cfDNA methylation status analysis	Methylation of cfDNA in Diagnosing and Monitoring Benign and Malignant Pulmonary Nodule	No
NCT05724264 (SOLSTICE)	Blood samples for biomarker detection	SingapOre Lung Cancer Screening Through Integrating CT With Other biomarkErs	No
NCT05699213	Blood samples for biomarker detection	A Pilot Study Evaluating the Feasibility of Novel MRI Sequences and Blood-Based Biomarkers for Discriminating Nodules Found During Lung Cancer Screening	No
NCT05766046(RISP)	Blood samples for miRNA analysis and biomarker detection	Early Diagnosis of Lung Cancer of the Italian Pulmonary Screening Network (RISP): Comparative Analysis for the Use of Low Dose Computed Tomography and Promotion of Primary Prevention Interventions in Subjects at High Risk for Lung Cancer	No
NCT05649046 (PREVALUNG ETOILE)	Blood samples and feces for microbiota analysis	Structuring of a Lung Cancer Screening Program Including Clinical, Radiological and Biological Phenotyping Useful for the Development of Individualized Risk Prediction Tools: PREVALUNG ETOILE	No
